# Response to the Letter by Matsubara

**DOI:** 10.31662/jmaj.2024-0186

**Published:** 2024-10-03

**Authors:** Masaki Mori

**Affiliations:** 1 Tokai University School of Medicine, Isehara, Japan

**Keywords:** Artificial Intelligence, Medicine, Machine Learning, Deep Learning, Neural Network

We appreciate the comment of Matsubara, emphasizing the need to separate the discussion of artificial intelligence (AI) from those related to writing assistance and those that aim to develop AI models. Indeed, these are very different aspects. I agree that most of the non-“AI-experts” will not aim to develop AI models but may use existing models to assist their writing. In addition, as the field develops, there will be cases in which the authors will report the outcome of using the established AI models. Articles describing these aspects will be welcome in the JMA journal. I would like to emphasize that those reporting external validations will be welcome, as this is increasingly recognized as an important portion of AI model development ^(1), (2)^.

For the regulation of the use of AI for writing assistance, the area is still in development, and thus, the appropriateness of the use should be evaluated on an individual bases. I agree that a standardized guideline will be helpful in identifying how AI could be used in the writing procedure, and there have been various attempts to develop such guidelines outside medicine. In the medical field, it has been reported that generative AI could be useful in some aspects, but may cause problems that were not recognized previously ^(3)^. I think that this should be an important topic in medicine and should be addressed in the near future.

The debate on how much assistance provided by AI for writing is acceptable is an interesting topic. I agree that clarifying the stance will be helpful in some cases, but in other cases, the “stance” may not be defined clearly enough. In my opinion, it is more important to clarify in the study, how much assistance was provided by generative AI so that the readers/reviewers could evaluate the value of the article correctly. The development of standards for citing the outputs of generative AI is also an important topic. These factors should also be included when developing guidelines.

The use of AI in medicine is an evolving topic that is being increasingly recognized as an important technology. I agree with Matsubara that we have just opened the door to a new scientific world and are looking forward to reading articles on medical AI in the JMA Journal.

## Article Information

### Conflicts of Interest

None

### Disclaimer

Masaki Mori is one of the Associate Editors of JMA Journal and on the journal’s Editorial Staff. He was not involved in the editorial evaluation or decision to accept this article for publication at all.
